# Use of a novel camelid-inspired human antibody demonstrates the importance of MMP-14 to cancer stem cell function in the metastatic process

**DOI:** 10.18632/oncotarget.25654

**Published:** 2018-06-29

**Authors:** Kuan-Hui E. Chen, Chuan Chen, Tyler Lopez, Kelly C. Radecki, Karissa Bustamante, Mary Y. Lorenson, Xin Ge, Ameae M. Walker

**Affiliations:** ^1^ Division of Biomedical Sciences, School of Medicine, University of California, Riverside, CA 92521, USA; ^2^ Department of Chemical and Environmental Engineering, University of California, Riverside, CA 92521, USA

**Keywords:** MMP-14, cancer stem cell, metastasis

## Abstract

Matrix metalloproteinases (MMPs) are considered excellent targets for cancer therapy because of their important roles in multiple aspects of tumor growth and metastatic spread. However, not all MMPs, or even all activities of specific MMPs, promote cancer. Therefore, there is a need for highly specific inhibitors. Monoclonal antibodies provide the potential for the degree of specificity required, but the isolation of antibodies able to inhibit a specific protease with high selectivity is challenging. Proteolysis specificity lies in recognition of the substrate in or around the active site, which generally forms a concave cleft inaccessible by human IgGs. Inspired by camelid antibodies, which have convex paratopes, we have produced a recombinant human IgG, designated 3A2, which binds in the substrate cleft of MMP-14, inhibiting its activity, but not the activity of highly homologous MMPs. In the 4T1 highly metastatic, syngeneic, orthotopic model of breast cancer, IgG 3A2 markedly inhibited growth of the primary tumor, but more importantly reduced metastatic spread to the lungs and liver by 94%. Stem cells in the tumor population expressed twice as much MMP-14 mRNA as bulk tumor cells. In addition to reducing dissemination of tumor stem cells, as would be expected from inhibition of MMP-14's ability to degrade components of the extracellular matrix, IgG 3A2 also inhibited the ability of individual stem cells to proliferate and produce colonies. We conclude that it is possible to produce antibodies with sufficient specificity for development as therapeutics and that IgG 3A2 has therapeutic potential.

## INTRODUCTION

Overwhelming evidence supports a critical role for proteases in cancer dissemination. Matrix metalloproteinases (MMPs) are a group of zinc-dependent endopeptidases that collectively degrade multiple components of the extracellular environment [1]. Degradation of the fibrous and proteoglycan components of the extracellular matrix is necessary for tumor growth and related angiogenesis, as well as the movement of metastasizing cells throughout the body. In addition to reducing physical barriers that limit tumor growth and cell dissemination, MMPs have complementary mechanisms through which they may promote cancer progression [2]. For example, the MMP may liberate a growth factor that attracts immune cells, which in turn produce chemokines that contribute to tumor cell survival and/or migratory ability [3]. MMPs are therefore considered potential therapeutic targets.

However, in addition to tumorigenic and pro-metastatic activities, MMPs are involved in embryonic development, mammary gland ductal branching, bone ossification, blood vessel remodeling, homeostasis of the extracellular environment, and control of innate immunity [4–9]. While many facets of proteolytic action are pro-tumorigenic, some MMPs exhibit tumor-suppressing effects under certain circumstances [10]. MMP-8 [11], MMP-3 [12] and MMP-12 [4, 13, 14] have demonstrated predominantly anti-tumor effects based on knockout models of cancer. It is therefore important that individual enzymes be targeted [3]. Prior attempts to utilize peptide or small molecule inhibitors of MMPs have all failed in clinical trials due to lack of specificity [15–17]. Antibody-based inhibitors are more specific and therefore promising, but generation of monoclonal antibodies (mAbs) that not only bind, but also inhibit proteases with high potency and selectivity is challenging. Recently, we demonstrated that incorporation of long, convex-shaped, camelid-like paratopes into human IgG enabled the antibody to target enzyme active pockets that are not accessible with conventional antibodies [18]. This resulted in the production of mAbs specific to a given MMP.

For a particular subclass of MMPs that are plasma membrane-spanning, evidence suggests an even greater array of activities. For example, while MMP-14 degrades collagens I, II and III, fibronectin, laminin, aggrecan and tenascin [1], and hence can clearly reduce physical barriers to tumor growth and metastatic spread, it is also a transmembrane protein capable of intracellular signaling [19]. Furthermore, MMP-14 association with another membrane protein, CD44, directs its localization to lamellopodia, the formation of which is important to cell migration and invasion [20]. MMP-14 also activates other pro MMPs such as proMMP-2/-9/-13, and through combined activities these MMPs cause effective tissue degradation and enhance tumor invasion and metastasis [21–23].

In the current study, we have used an engineered human mAb that specifically inhibits MMP-14 and determined its potential therapeutic effect in a highly metastatic, syngeneic mouse model of breast cancer. In addition to a 94% suppression of metastasis to the lungs and livers of animals treated with the mAb, we found higher expression of MMP-14 by cancer stem cells and evidence that MMP-14 contributes to the ability of cancer stem cells to initiate a metastatic colony.

## RESULTS

### IgG 3A2 was a potent and selective inhibitor of human and murine MMP-14s

**Figure 1:**
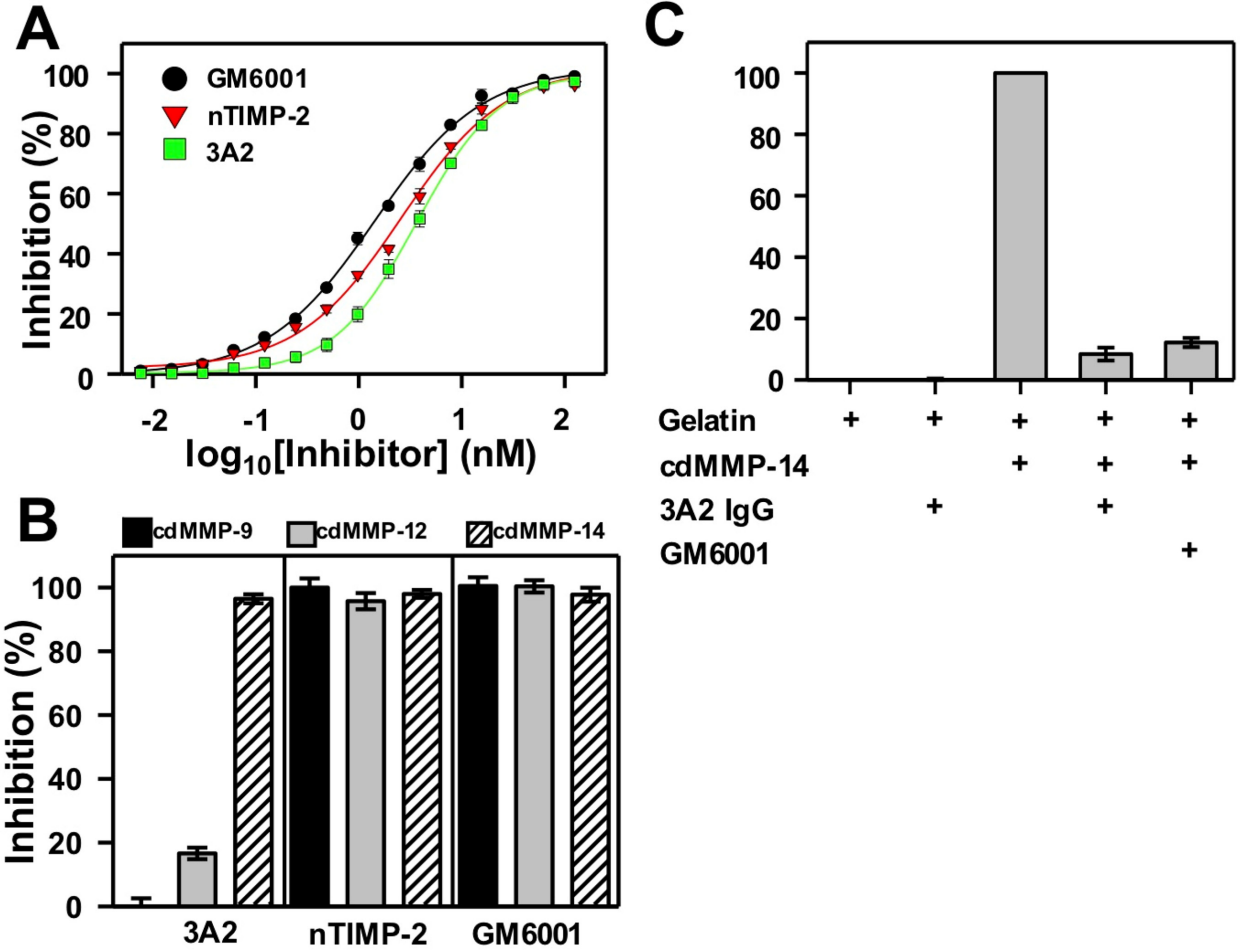
Biochemical characterizations of IgG 3A2 **(A)** Dose-inhibition curves. 1 nM human cdMMP-14 and 1 μM FRET peptide substrate, M-2350, were used. **(B)** Inhibition selectivity. 10 nM human cdMMP-9, cdMMP-12, or cdMMP-14 were reacted with 62.5 nM inhibitor for 30 min before the addition of 1 μM M-2350. **(C)** Gelation hydrolysis tests. 500 nM human cdMMP-14 was reacted with gelatin in the presence or absence of 1 μM inhibitor and characteristic degradation bands were quantitatively analyzed via SDS-PAGE. (n=3) Data are presented as the mean ± S.D.

We have previously reported the binding kinetics and specificity of Fab 3A2 [18], but development of the whole IgG required reevaluation since IgG molecules generally have an increased affinity and the increased bulk may have changed specificity. IgG 3A2 was produced in 293F cells, purified from culture medium by affinity for protein A, with typical yields of 35 mg purified IgG (purity >98%) per liter culture medium. The purity of 3A2 IgG was determined by SDS-PAGE (Supplementary Figure 1). The catalytic domain (cd) of MMP-14 was purified from *E. coli* periplasm, as described previously [24]. A purity > 95% was confirmed by SDS-PAGE. Binding kinetics of 50-200 nM IgG 3A2 to immobilized human cdMMP-14 were analyzed using bio-layer interferometry. An average kinetic association coefficient (k_on_) of 9.5×10^6^ M^-1^·s^-1^ and an average kinetic dissociation coefficient (k_off_) of 3.6×10^-2^ s^-1^ were determined, allowing calculation of the equilibrium dissociation constant (K_d_) equal to 3.8 nM (Table 1). Inhibition potency of IgG 3A2 was measured with 1 nM human cdMMP-14 and 1 μM peptide substrate. The inhibition IC_50_ of IgG 3A2 against human MMP-14 was 3.8 nM, which is comparable to its native inhibitor nTIMP-2's IC_50_ of 3.0 nM under the same reaction conditions, and the potent small molecule inhibitor GM6001's IC_50_ of 1.5 nM (Figure 1A). Importantly, 3A2 showed a therapeutically-desired selectivity towards cdMMP-14 over highly homologous cdMMPs -9 and -12. At 62.5 nM, IgG 3A2 completely inhibited (97%) activity of 10 nM cdMMP-14, while there was no inhibition towards cdMMP-9 under the same conditions and a weak inhibition (16%) towards cdMMP-12 (Figure 1B). In contrast, and as expected, neither nTIMP-2 nor GM6001 exhibited selectivity, with 95-100% inhibition for each of the three MMPs tested. The ability of IgG 3A2 to inhibit gelatinolytic activity of human cdMMP-14 was determined by incubating cdMMP-14 with gelatin in the presence or absence of inhibitor and digitizing the densitometry of produced characteristic bands on SDS-PAGE. Compared to the degree of gelatin hydrolysis with cdMMP-14, IgG 3A2 inhibited 90% activity of cdMMP-14 after a 24 hr incubation, while the same concentration of GM6001 gave 87% inhibition (Figure 1C). This result was consistent with a previous study showing that mAb 3A2 blocked cdMMP-14 from hydrolyzing type I collagen [18], a physiological substrate relevant to cancer metastasis *in vivo* [25].

**Table 1:** Binding affinity and inhibition potency of IgG 3A2

	k_on_ (1/Ms)	k_off_ (1/s)	K_D_ (nM)	Inhibition IC_50_ (nM)
**with human cdMMP-14**	9.5×10^6^	3.6×10^-2^	3.7	3.8
**with murine cdMMP-14**	9.9×10^4^	6.8×10^-4^	6.9	7.9

Determination of binding affinity and inhibition potency of IgG 3A2 showed a similar K_D_ and inhibition IC_50_ when interacted with human and murine cdMMP14s. Therefore, the IgG 3A2 was a potent and selective inhibitor of both human and murine MMP-14s.

To test whether IgG 3A2 interacted with murine cdMMP-14 (97% amino acid identity to human cdMP-14), binding kinetics and inhibition potency were measured (Table 1). The k_on_ with murine cdMMP-14 was 9.9 × 10^4^ M^-1^·s^-1^, which was significantly lower than the k_on_ with human cdMMP-14. However, once bound, IgG 3A2 binding was tighter to the murine version with a slow k_off_ of 6.81 × 10^-4^ s^-1^. Overall, IgG 3A2 exhibited a dissociation constant (K_d_) of 6.9 nM to murine cdMMP-14. Similarly, inhibition potency IC_50_ of IgG 3A2 against murine cdMMP-14 was measured as 7.9 nM. Therefore, IgG 3A2 interacts with both human and murine cdMMP-14.

### Pharmacokinetic analysis of IgG 3A2 *in vivo*

**Figure 2:**
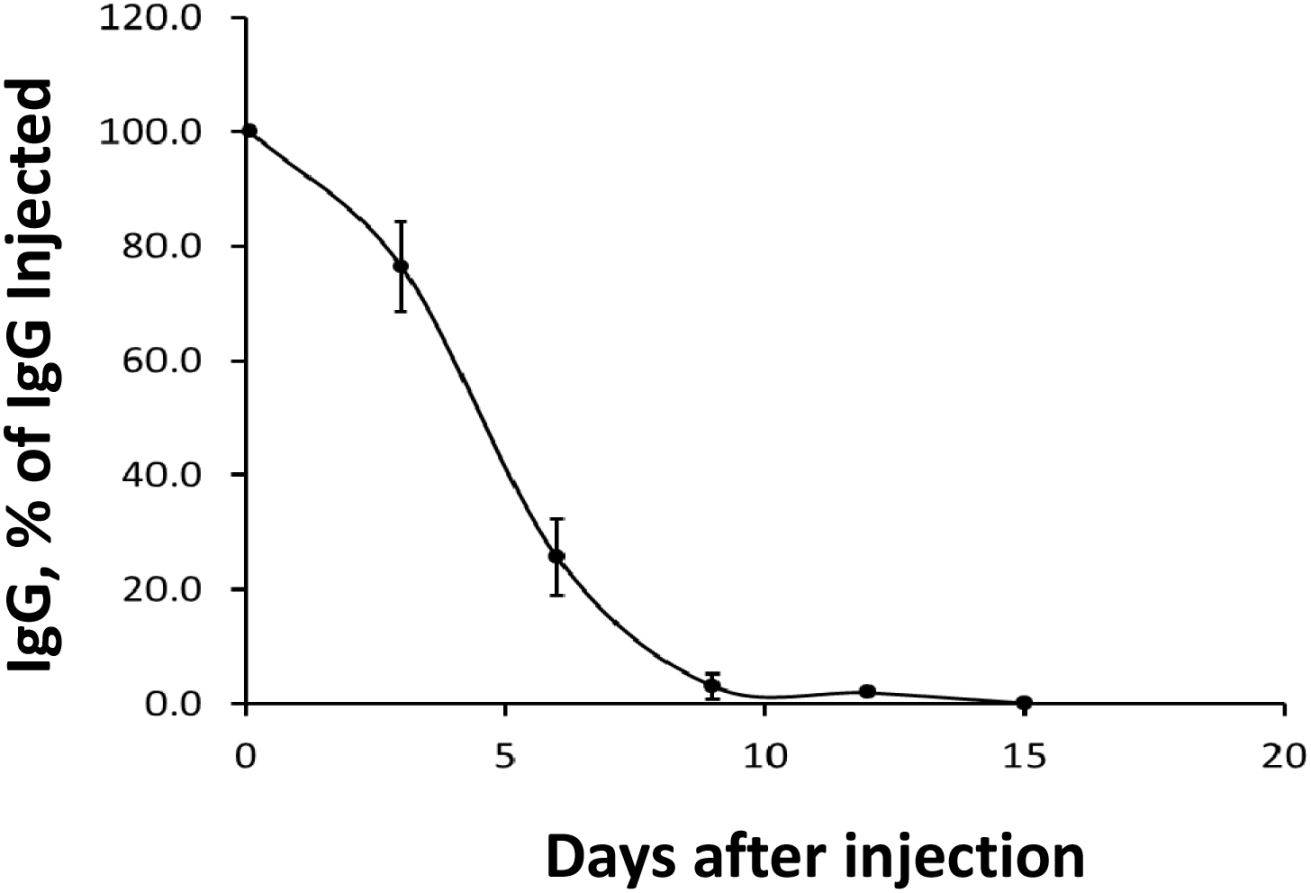
Disappearance of IgG 3A2 from mouse serum 20 μgof IgG 3A2 was injected into the tail vein and samples of plasma were taken at the times indicated for measurement of human IgG by ELISA. The determined half-life of IgG 3A2 was 4.8 days. (n=4) Data are presented as the mean ± S.E.M.

To determine the potential of using IgG 3A2 as a future therapeutic, its *in vivo* half-life was examined following a bolus injection. The amount of IgG 3A2 present in blood 2 hours after injection was considered the initial circulating concentration (100%). The relative amounts of IgG 3A2 dropped to 75.5%, 24.3%, 5.9%, 4.5% and 0.5% at days 3, 6, 9, 12 and 15, respectively (Figure 2), giving a half-life of ~4.8 days, similar to that of serum immunoglobulins in adult mice [26, 27].

### Administration of IgG 3A2 repressed both primary tumor burden and metastasis in the highly metastatic mouse 4T1 model

**Figure 3:**
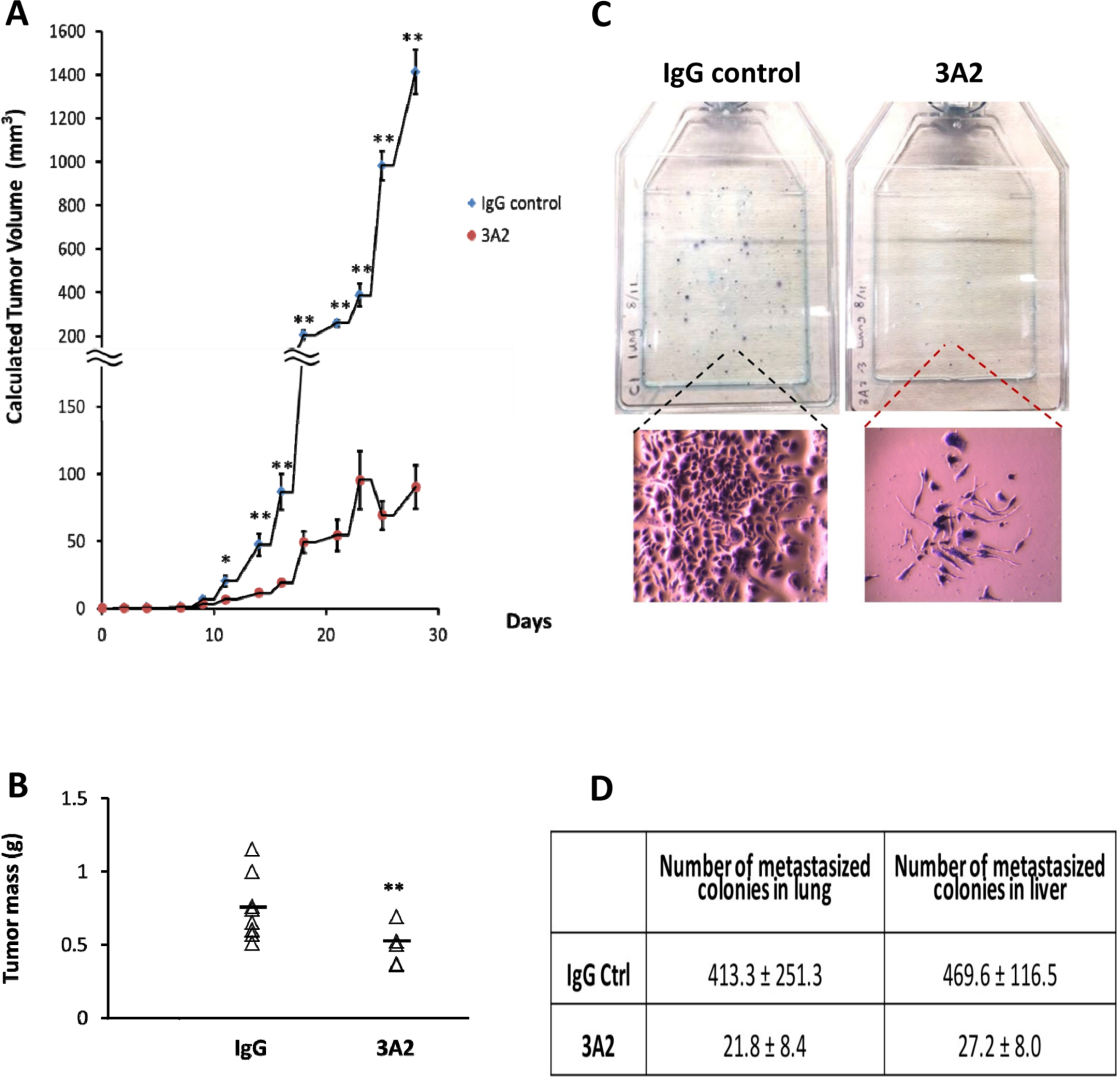
IgG 3A2 treatment significantly reduced both primary tumor burden and metastasis The primary tumor burden was assessed by **(A)** tumor volume (n=11 in both control IgG and IgG 3A2 group) and **(B)** tumor weight (n=8 in control IgG and n=6 in IgG 3A2 group). Data are presented as the mean ± S.D.; ^*^P < 0.05 and ^**^P < 0.01. **(C)** Metastasis with control IgG or IgG 3A2 treatment was determined using the 6-thioguanine selection method. Each colony formed after 6-thioguanine selection is derived from one metastatic 4T1 cell. 6-thioguanine selection of metastatic cells in the lung is illustrated and the relative size of a typical metastatic colony in the two treatments is shown below. **(D)** Number of metastasized colonies in lung and liver.

Three thousand highly metastatic, murine, syngeneic mammary carcinoma 4T1 cells were injected orthotopically into FoxP3-GFP Balb/c mice to establish a single primary tumor per mouse (N=11 for each group). IgG 3A2 or control IgG were intraperitoneally administered at 100 μg per mouse every two days for 30 days. At this dosage, IgG 3A2 significantly reduced primary tumor burden, as assessed by volume calculated from caliper measurements (Figure 3A) and by dissected tumor weight at day 30 (Figure 3B). While there was a highly significant negative impact of 3A2 on tumor growth using both measures, the difference between the two modes of assessment highlights inaccuracies in both methods. Although IgG 3A2 reduced the size of the primary tumor, the rate of proliferation of what tumor cells were present was unaltered, as assessed by the incorporation of EdU (Supplementary Figure 2). General assessments of animal health showed no effects of IgG 3A2 administration on body weight (Supplementary Figure 3), movement, fur condition or food consumption.

Since MMPs participate in cell invasion [28], we next investigated whether treatment with IgG 3A2 affected metastasis. Because 4T1 cells are resistant to 6-thioguanine while normal somatic mouse cells are not, production of a single cell suspension and incubation in 6-thioguanine selects for metastatic cells within tissues. Individual metastatic cells then grow into colonies. As illustrated in Figure 3C and quantified in Figure 3D, mice that were treated with IgG 3A2 for 30 days had significantly fewer metastatic cells in both lungs and liver, representing 94.7% and 94.2 % metastasis reduction, respectively (Figure 3D). In addition, most colonies were also significantly smaller in diameter (Figure 3C), demonstrating MMP-14 inhibition had a long-term effect on the proliferation of metastatic cells since each colony grows from a single cell present on day 1 and no extra antibody was added during the 7-day 6-thioguanine selection period.

### 3A2 treatment enhanced immune cell activity within the tumor microenvironment

**Figure 4:**
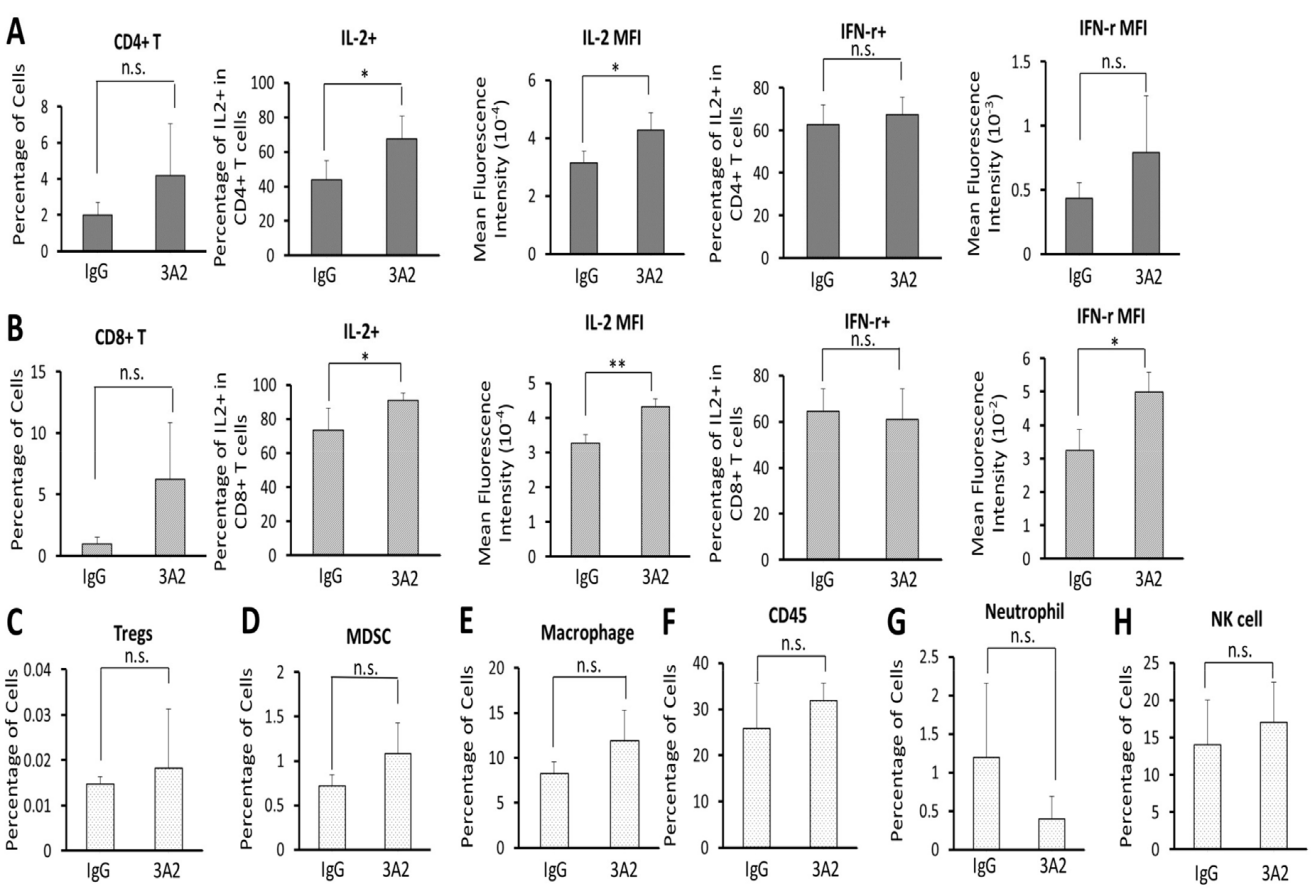
IgG 3A2 treatment did not affect immune cell composition of the primary tumor, but did result in increased T cell activation The percentages of immune effector cells: CD4^+^ T cells **(A)** and CD8^+^ T cells **(B)** and immune suppressor cells: T regulatory cells (Tregs) **(C)**, myeloid derived suppressor cells (MDSC) **(D)**, macrophages **(E)**, CD45 cells **(F)**, neutrophil **(G)** and NK cells **(H)**. The activity of CD4^+^ T cells and CD8^+^ T cells was further determined by the percentage of these cells expressing IL-2 and IFN-γ and mean expression intensity per cell (A and B). (n=11 in A-E and n=3 in F-H) Data are presented as the mean ± S.D.; ^*^P < 0.05 and ^**^P < 0.01.

A reduced primary tumor size could have resulted from an increase in immune clearance of tumor cells, which in turn could have been mediated by a decrease in immune suppressive cells or an increase in immune effector cells. In addition, most tumor stromal cells, including tumor associated macrophages, endothelial cells and tumor fibroblasts are known to express MMP-14 and changes in their number within the tumor may have contributed to reduced tumor growth. This is particularly true of macrophages since 4T1 tumors at 30 days grown from a 3000 cell inoculate are approximately 10% macrophages (see part of Figure 4).

As shown in Figure 4A, there was no effect of 3A2 on the overall percentage of CD4^+^ T cells present in the tumor microenvironment. However, although the percentage didn't change significantly, the proportion that were IL-2^+^ increased, as did the expression of IL-2 per cell. There was no difference in the proportion of CD4^+^ T cells expressing IFN-γ or the level of expression of IFN-γ per CD4^+^ T cell as a result of 3A2 treatment.

Similar to CD4^+^ T cells, there was no effect on CD8^+^ T cell percentages, although there was a trend towards an increased percentage in the 3A2 treatment group (Figure 4B) and a higher percentage of CD8^+^ T cells produced IL-2 with a higher expression level of IL-2 per CD8^+^ T cell (Figure 4B). Furthermore, with CD8^+^ cells there was also increased production of IFN-γ per CD8^+^ T cell. Given that there was no increase in the very small percentage of cells that were Tregs (Figure 4C), these data are consistent with increased activation of T helper cells and activation of cytotoxic CD8^+^ cells.

An additional cell population, generally identified as immune suppressive, is myeloid- derived suppressor cells (MDSC, cd11b^+^Gr1^+^). As shown in Figure 4D, there was no change in this population. Macrophages can be stimulatory or suppressive, depending on phenotype. The overall percentage of tumor macrophages was not affected by IgG 3A2 (Figure 4E). However, there is an ~54%, but not statistically significant, reduction in tumor infiltrating macrophages (F4/80) that were of the M2 phenotype (F4/80^+^iNOS^-^CD206^+^CD36^+^, Supplementary Figure 4). It is possible therefore that there may be some increase in the M1/M2 ratio by IgG 3A2 treatment. There were also no significant effects on other immune cell populations, including CD45^+^ T cells (Figure 4F), neutrophils (cd11b^+^Ly6G^+^CD62L^+^) (Figure 4G) and NK T cells (NK1.1^+^) (Figure 4H) although a trend towards a reduction in neutrophils by IgG 3A2 treatment was observed. Taken together, the results indicate that 3A2 had little effect on the immune cell composition of the tumors present, but, based on the increased activation of T cells, that immune clearance may well have contributed to the reduced primary tumor burden seen with 3A2 treatment.

### Targeting MMP-14 in cancer stem cells suppressed their sphere-forming capacity and proliferation

**Figure 5:**
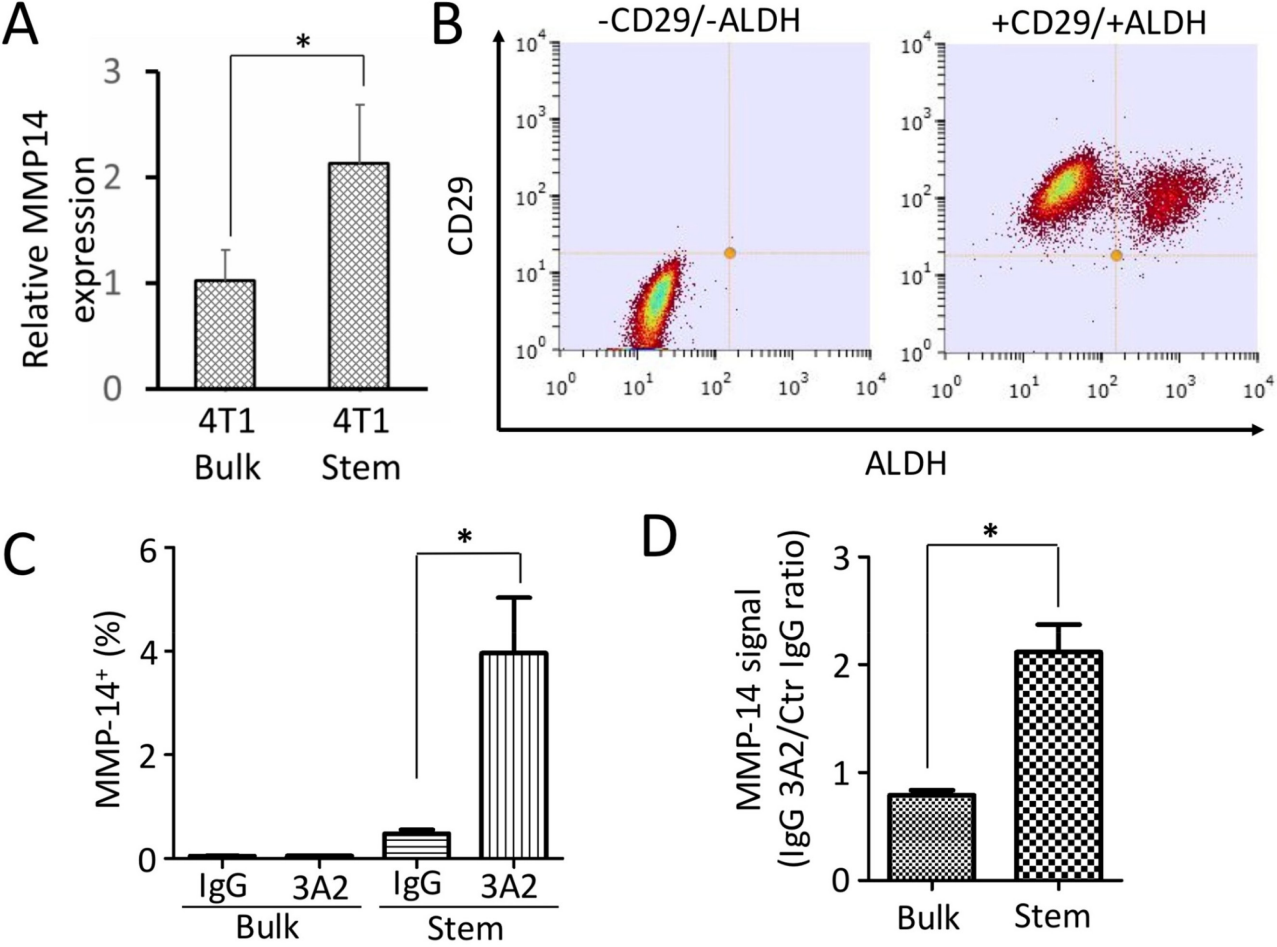
Expression of MMP-14 and potential binding difference of IgG 3A2 to MMP-14 between cancer stem cells and bulk tumor cells **(A)** MMP14 mRNA expression in bulk tumor cells (left) compared to stem cells (right) derived from bulk tumor cells. **(B)** CD29^+^ and CD29^+^/ALDH^+^ cells. **(C)** Statistics of MM-14 positive cells. **(D)** Relative expression levels of MMP-14 on surface of bulk and stem cell populations. Data are presented as the mean ± S.D. from three trials; ^*^P < 0.05.

Although bulk tumor cells can metastasize, it is the tumor stem cells from within the population that result in a metastatic colony [29]. MMP-14 is well known to be important in invasion, but we did not know whether tumor stem cells expressed MMP-14. A comparison of mRNA expression in bulk 4T1 (contains some stem cells) with expression in derived stem cells is shown in Figure 5A. Stem cells express 2-fold the level in bulk tumor cells. To determine whether this was true at the protein level and to determine whether IgG 3A2 recognized murine MMP-14 when present in the membrane, flow cytometry was performed. To distinguish cancer stem cells from the bulk cells, 4T1 cells were labelled with anti-mouse CD29 and treated with AldeFluor. Results indicate that >99±0.1% of 4T1 cells were CD29^+^ and 21±5% were ALDH^+^ (Figure 5B). The CD29^+^/ALDH^+^ populations were next labelled with IgG 3A2 and anti-human IgG-PE. Polyclonal human IgG was used as the negative control. As shown in Supplementary Figure 5 and Figure 5C, 4.0±1.8% of CD29^+^ALDH^+^ cells were stained positively for MMP-14 while only < 0.1% bulk cells were MMP-14^+^. For the stem cells, the average fluorescence signal of MMP-14 was 2.1(±0.4)-fold higher when stained with IgG 3A2 group versus control IgG, while there was no significant difference for bulk cells (Figure 5D).

**Figure 6:**
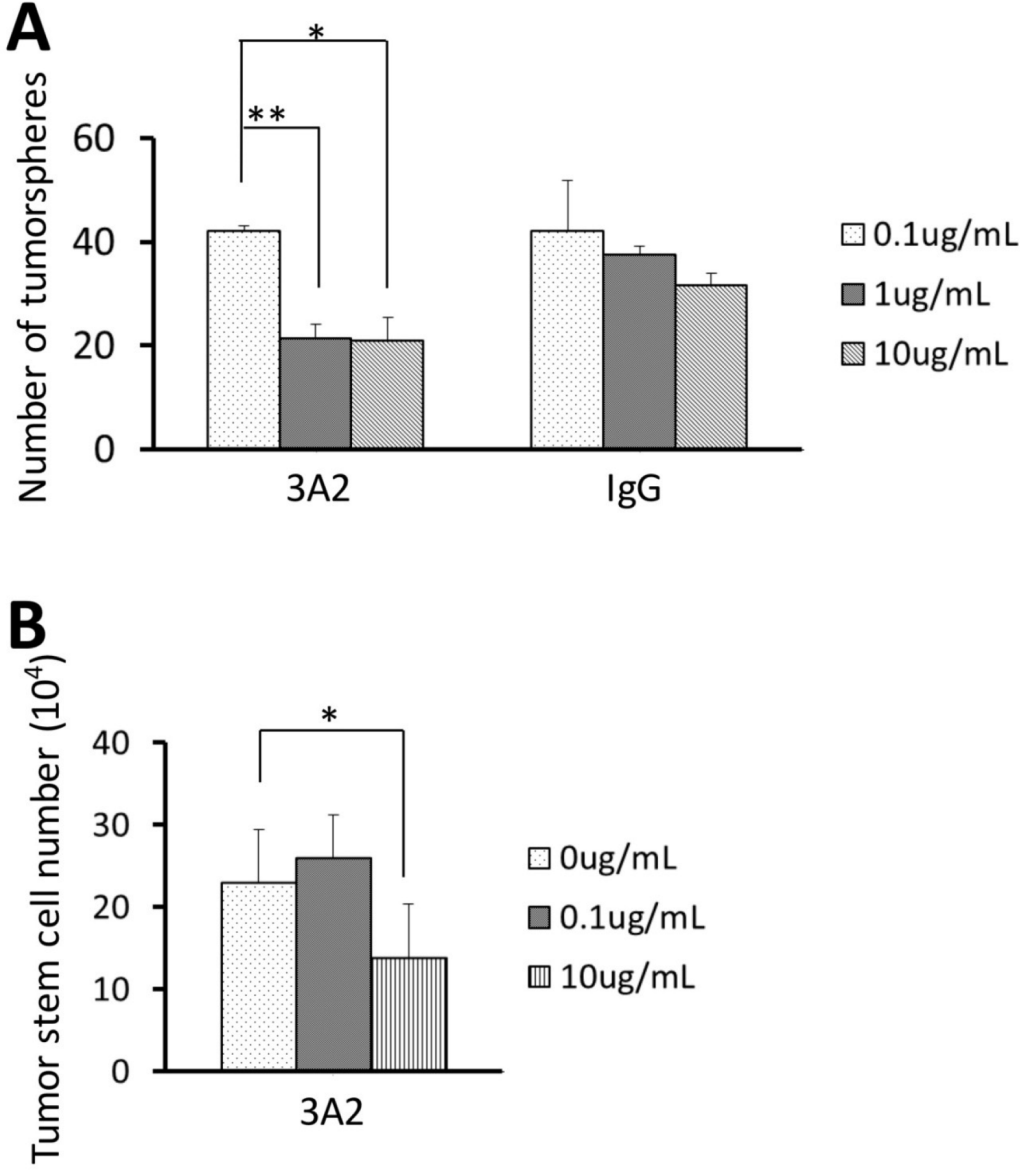
Effect of IgG 3A2 on tumorsphere formation and cancer stem cell proliferation **(A)** The effect of IgG 3A2 treatment on stem cells tested their sphere forming ability, and **(B)** proliferation which was verified by direct cell counting. n=3 in both (A) and (B). For proliferation, the number of cells from each repeat was derived from an average of 3 wells. Data are presented as the mean ± S.D.; ^*^P < 0.05 and ^**^P < 0.01.

Since the metastatic colonies formed after 3A2 treatment were not only fewer in number, but also smaller (Figure 3C), we determined whether targeting MMP-14 in tumor stem cells would affect the ability of tumor stem cells to form tumor spheres *in vitro* where expansion of a growing tumor would be much less dependent on remodeling of the extracellular matrix. As shown in Figure 6A, addition of 3A2 had an inhibitory effect on sphere formation compared to the control antibody. We therefore questioned whether this reduction in sphere formation was due to inhibitory effects on cell proliferation or a change in the stemness. When tumor stem cells were seeded in 6 well plates and grown in the presence of different concentrations of IgG 3A2, there was a reduced number of tumor stem cells at the highest concentration tested (10 μg/mL) (Figure 6B). However, there were no effects on stemness, as assessed by expression of *oct4* and *sox2* (Supplementary Figure 6).

When cells migrate, their phenotype changes from epithelial to mesenchymal in nature. To further explore the effect of targeting MMP-14 on tumor stem cells, we analyzed the expression of mesenchymal markers. There were no significant effects on *E-cadherin, fibronectin, vimentin, twist-1, or snail-2* mRNA expression, although snail-2 showed a trend towards reduced expression with IgG 3A2 treatment (Supplementary Figure 7). This is likely because the stem cells are already mesenchymal in nature. Together with the results of the 6-thioguanine assay following *in vivo* 3A2 IgG treatment, the results support a role for MMP-14 in the establishment and growth of stem cell-derived colonies and inhibition of this role by 3A2.

## DISCUSSION

In the last two decades, numerous MMP small molecule or peptidomimetic inhibitors have been tested for cancer treatments [15–17]. However, all these inhibitors failed in clinical trials due to lack of efficacy and severe side-effects such as musculoskeletal pain and inflammation [30]. Apart from dose-limiting side-effects, the other main reason for the failure of MMP inhibitors in phase III clinical trials is the broad-spectrum nature of these original drugs [17]. The majority of MMP inhibitors tested clinically were derivatives of hydroxamic acid, which potently chelate zinc and many other transition metals that serve as co-factors for a plethora of enzymes, among other important activities. The musculoskeletal pain and inflammation observed clinically was most likely due to the lack of specificity and the myriad of reactions affected by the inhibitor. It thus became evident that inhibiting MMPs nonspecifically was not desirable [15, 16]. In our previous studies, a panel of Fabs inhibiting MMP-14 had been generated from synthetic antibody libraries carrying convex paratopes [18]. Particularly, Fab 3A2 exhibited nM inhibition potency and exclusive selectivity toward MMP-14. Biochemical characterizations of 3A2 demonstrated that it was a competitive inhibitor [18, 31], and able to prevent MMP-14 from activating proMMP-2 [18]. Tests with a melanoma xenograft mouse model suggested that administration of Fab 3A2 significantly reduced cancer spread [31]. Although the use of Fab fragments as therapeutics has several advantages, their fast clearance rate (35 times faster than whole IgG) necessitating more frequent dosing intervals [32], is less desirable for patients and markedly increases cost. In the current study, we therefore produced the whole 3A2 IgG with >98% purity to test *in vivo* stability and the effects of IgG 3A2 on cancer burden relief in a syngeneic mouse breast cancer model with extremely fast and spontaneous metastasis. This model also allowed us to examine for the first time the impact of IgG 3A2 on complete immune responses to tumor cells, on cancer stem cell function, and effectiveness against metastatic spread in a naturally, rapidly metastasizing model. In comparison to the Fab 3A2, which had an inhibition potency (IC_50_) of 9.7 nM [18], the IgG 3A2 has significantly improved inhibition potency (IC_50_ 3.8 nM) comparable to the natural inhibitor nTIMP-2 (IC_50_ 3.0 nM) and the small molecule inhibitor, GM6001 (IC_50_ 1.5 nM) along with greater specificity compared to the small molecules for MMP-14 inhibition.

Clearance from the circulation after a bolus injection occurred with a half-life of 4.8 days. With the assumption that the clearance rate of continuous IgG 3A2 treatment was the same as the first bolus, it will take 9 days to reach a consistent concentration at ~220 μg/mL plasma in the animals if 100 μg IgG 3A2 is given every 2 days. This concentration injected is lower than has been used for efficacy studies of other monoclonal antibodies against MMP-14 [33–35] and yet there was a highly significant impact on both primary tumor growth and, more importantly, on metastatic spread. We therefore have evidence that this particular antibody could be an effective and cost efficient therapeutic.

Several factors could have contributed to the reduction in primary tumor size. One potential factor could have been a reduction in the relative number of immune cells capable of migrating into the tumor, especially since macrophages constitute around 10% of the tumor at 30 days. However, our results did not find any effect on the relative percentages of any immune cell populations, including macrophages, myeloid-derived suppressor cells, Tregs and T effector cells. The change in tumor mass is therefore not related to the relative number of immigrating cells.

An inhibition of primary tumor growth could also have been related to increased immune killing of tumor cells. Consistent with immune cell killing playing a role in reduced tumor size is the observed activation of T helper cells and cytotoxic T cells. Given that there was no change in the proportion of immune suppressive cells present, the mechanism responsible for increased activation of T effector cells with MMP-14 inhibition is unclear. Interestingly, there is evidence that CD4 effector T cells express lower MMP-14 upon activation [36], but this has so far been presumed to be a consequence of activation and the lack of continued need for migration. Most likely, inhibition of MMP-14 activity prevented the liberation of suppressive cytokines bound to the extracellular matrix such as TGFβ [37], but this remains to be determined.

In addition to the enhanced immune killing, a reduction in tumor size could have been brought about by reduced cell proliferation. However, for the cells that were present in the differently sized primary tumors, there were no effects of IgG 3A2 treatment on bulk tumor cell proliferation, as examined by EdU incorporation.

Since the percentage of cancer stem cells in the tumor mass is small, we would not expect to be able to discern an effect specifically on stem cells when measuring whole tumor proliferation by EdU incorporation. However, the *in vitro* results with the stem cell population show that IgG 3A2 can inhibit colony formation and the total number of cells at a dose of 10 μg/ml. If our calculations on the steady state amount present in the animals is correct, there would have been sufficient administered antibody to do the same *in vivo*. Although all epithelial cells in the tumor have the capacity to divide, an inhibition of stem cells would certainly contribute to reduced tumor growth. A stimulatory effect of MMP-14 on cell proliferation [38, 39] has been shown to be mediated through its hemopexin domain [19, 20], but whether a similar effect of MMP-14 occurs in cancer stem cells has not been previously addressed. Our results show that inhibition of MMP-14 inhibits stem cell proliferation. Furthermore, that the effect of 3A2 treatment was long lived since it persisted after the cells were *in vitro* and removed from exposure to the antibody during the 7-day 6-thioguanine selection.

Although a reduction in the size of the primary tumor is good, the most dramatic and potentially clinically-relevant outcome of treatment with anti-MMP-14 IgG 3A2 was a 94% inhibition of metastasis to the lungs and livers. Like the effect on primary tumor growth, there are a number of possible mechanisms. One is a decrease in primary tumor size and thereby a decrease in cells available for metastasis. While this may have contributed, initiation of metastasis has been demonstrated to have little relationship to the size of the primary tumor [40]. In fact, this is one reason why screening of anti-cancer drugs based on their ability to reduce the size of the primary tumor has not been a reliable measure of the eventual utility of the drug in the clinic [40].

The metastatic process is complex and differs somewhat in different types of cancer, but migration of cells must employ some level of protease activity to digest a path through/around both fibrous and proteoglycan elements of the extracellular matrix. For 4T1 tumors, metastasis is at least primarily the result of migration of stem cells [41]. Given the known functions of MMP-14, it seems reasonable to suggest that inhibition of protease activity reduced the ability of stem cells to migrate from the primary tumor into the blood stream and from the blood stream into the stroma of the lungs and liver. Consistent with this premise is the higher expression of MMP-14 in and on cancer stem cells versus bulk tumor cells. To our knowledge, this has not been previously reported. Consistent with reduced metastasis by IgG 3A2 treatment, is the trend towards reduced expression of the mesenchymal marker, *Snail*, in cancer stem cells. An MMP-14/CD44/Snail axis has been previously identified as an important regulator of tumor invasion in other tumors [42].

In summary, the data show it is possible to produce a potent recombinant human IgG that specifically inhibits MMP-14 and not other highly homologous MMPs. Furthermore, that IgG 3A2 markedly inhibits metastasis. In addition to likely inhibitory effects on migration, IgG 3A2 inhibits stem cell proliferation and colony formation, indicating an important role for MMP-14 beyond simple inhibition of proteolytic degradation of the extracellular matrix.

## MATERIALS AND METHODS

### Production of MMPs and TIMPs

Human catalytic domains (cd) of MMPs-9, -12 and -14, murine cdMMP-14, human N-terminal domain of tissue inhibitor of metalloproteinase-2 (nTIMP-2), each containing 6×His tags at their C-termini were produced by culturing transformed BL21 *E. coli* cells in 2×YT broth in the presence of 0.1 mM isopropyl β-D-1-thiogalactopyranoside at 30°C for 15 hours, as described previously [43, 44]. After expression, periplasmic fractions were prepared by osmotic shock with 25% sucrose followed by treatments with lysozyme and water. Periplasmic solutions were clarified by centrifugation at 15,000 × *g* for 15 minutes at 4°C, passed through a 0.22 μm filter and purified by affinity chromatography using Ni-NTA agarose (Qiagen). Purified protein samples were buffer-exchanged into 50 mM Tris-HCl pH 7.5, 150 mM NaCl, 5 mM CaCl_2_, 0.5 mM ZnCl_2_ by dialysis at 4 °C using SnakeSkin tubing (Fisher), and concentrated by ultrafiltration with MWCO of 10 kDa (Amicon). The purity and concentration of produced recombinant proteins were determined by SDS-PAGE and OD_280_ absorption measurements.

### Production of IgG 3A2

3A2 V_H_ and V_L_ genes [18] were PCR purified and cloned into IgG expression vectors, which carry a CMV promotor with an intron and Woodchuck posttranscriptional regulatory element in addition to the antibody gene. The constructed vectors for heavy chain and light chain were mixed at a molar ratio of 1:1 for a total of 10 μg/mL and incubated with 30 μg/mL polyethyleneimine (PEI) HCl MAX (MW=40,000, Polysciences) in Opti-MEM^TM^ (ThermoFisher) medium for 10 min at room temperature. Then the DNA/PEI mixture were added into a 9-fold volume of 293F cells (3.0 × 10^6^ cells/mL with viability > 98%) in Expi293 medium (ThemoFisher). The transfected cells were cultured in round bottles at 135 rpm, 37 °C, 8% CO_2_ incubator for 7 days. Culture medium was clarified by centrifugation, and IgG 3A2 was purified by protein A affinity chromatography (GenScript) following the manufacturer's protocol. The concentration of purified IgG 3A2 was determined by SDS-PAGE and OD_280_ absorption measurements.

### Fluorescence resonance energy transfer inhibition assays

1 μM IgG 3A2, nTIMP-2, or the small molecule inhibitor, GM6001 (Millipore), was serially 2-fold diluted into assay buffer, and incubated with 1-10 nM human cdMMP-14 for 30 min at room temperature. The kinetic measurements were started with the addition of 1 μM M2350 peptide substrate (Bachem) and the fluorescence was monitored with excitation and emission wavelengths at 325 and 392 nm, respectively, using a fluorescence plate reader (BioTek). Inhibition percentages at given concentrations were calculated by comparing the initial slopes with and without the inhibitor. IC_50_ was determined as the concentration resulting in 50% inhibition. Inhibition assays for human cdMMP-9 and cdMMP-12 were also conducted to test selectivity.

### Gelatin hydrolysis studies

500 nM human cdMMP-14 was incubated with 1 mg/mL gelatin (porcine skin, Sigma) in the absence or presence of 1 μM inhibitors for 24 hours at room temperature, then samples were analyzed by 12% SDS-PAGE. Gelatin mixed with cdMMP-14 in the absence of inhibitor was used as a negative inhibitory control, and the background hydrolysis without cdMMP-14 was also tested. The degree of gelatin hydrolysis was quantified by densitometry of produced characteristic bands using Image Lab.

### Affinity measurements

Binding kinetics of IgG 3A2 towards human cdMMP-14 were analyzed by bio-layer interferometry using ForteBio BLItz. Purified cdMMP-14 was biotinylated using an EZ-link sulfo-NHS-LC-biotin labeling kit (Thermo Scientific), and loaded onto a streptavidin biosensor for 120 sec. The coated biosensor was incubated in 50 mM HEPES to establish baselines. IgG 3A2 was then introduced at 40-800 nM, the association to immobilized cdMMP-14 was monitored for 2 min, and then the IgG was allowed to dissociate in 50 mM HEPES (pH 6.8) for 2 min. Determined k_on_ and k_off_ were used for K_D_ value calculation. The affinity of IgG 3A2 towards murine cdMMP-14 was measured by loading 50 nM IgG 3A2 onto a protein A biosensor, then the association and dissociation of 50-200 nM murine cdMMP-14 was monitored using the same protocol.

### Tumor initiation and pharmacokinetic analysis

All animal experiments were conducted under University of California Riverside IACUC approved protocols.

Balb/c mice (Jackson Laboratories) were maintained as a breeding colony. For some experiments, Balb/c mice that express GFP under the control of the Foxp3 promoter (Jackson Laboratories) were used in order to be able to determine Treg cells in live preparations. Eight-week-old female mice were used in all experiments. Mouse syngeneic breast cancer 4T1 cells were maintained in complete media (RPMI-1640 medium (ATCC) with 10% FBS (Gibco)) at 37°C in a 5% CO_2_ and 95% humidity incubator and used within 15 generations. Three thousand 4T1 cells were mixed with 50 μl Matrigel (BD Biosciences) and orthotopically injected into the fourth mammary fat pad. At the same time, all mice were intraperitoneally given either 100 μg IgG 3A2 or human polyclonal control IgG (Sigma). Mice (N=11 each group) continued to receive IgG 3A2 or control IgG treatment every 2 days until sacrifice. Typically, with this number of cells, a palpable mass at the mammary fat pad can be appreciated 7-10 days after the initial tumor cell injection. Tumor sizes were measured by caliper and tumor volumes were calculated using the equation x^2^y/2, where x and y indicate the width and length, respectively [45]. All mice were sacrificed on day 30 and immune composition within the tumor microenvironment was analyzed by flow cytometry.

For pharmacokinetic analysis, purified IgG 3A2 was exchanged from 50 mM HEPES, 150 mM NaCl, pH 6.8 to Dulbecco's PBS (DPBS) using Centricon 10 microcentrifuge tubes (14,000 ×*g* for 20 min at 4°C, 4 times). 20 μg IgG 3A2 was injected into two mice via the tail vein and clearance was monitored by obtaining 50 μl serum tail vein samples from 2 hours to 12 days. The disappearance of IgG 3A2 from mouse serum was determined using a human IgG ELISA kit (Sigma) according to the manufacturer's instructions. Assays were carried out using 3 dilutions at each time point (in duplicate). Additionally, mouse serum was tested for cross reactivity between the mouse IgG in mouse serum and ELISA captured antibody and interference signals generated by non-specific binding of compounds in mouse serum by assaying alone (at 1/20, 1/100, 1/1000, and 1/10,000-fold dilutions) and when added to a control human IgG sample (at 1/20, 1/100, and 1/1000-fold dilutions). There was no cross-reactivity.

### EdU staining

The nucleoside analog, 5-ethynyl-2-deoxyuridine (EdU), was injected intraperitoneally (25 μg/g body weight) 4 hours before tissue harvesting. Incorporated EdU was detected using the Click-IT™ system (Invitrogen) and analyzed by flow cytometry (BD Canto II).

### Quantification of tumor metastasis

Lung and liver tissues from IgG 3A2- or control IgG-treated mice were minced by blade and digested with accutase (Innovative Cell Technologies) for 5 min at 37°C. After allowing tissue remnants to settle, supernatants were transferred and cultured in 75 cm^2^ flask in complete media with 60 μM 6-thioguanine. Metastatic colonies, formed after the 7-day selection period, were fixed with methanol for 5 min, washed with DPBS 3 times and stained with 0.03% (w/v) methylene blue for 5 min. Each metastatic blue colony was then counted [46].

### FACS analyses

4T1 cells, cultured as described above, were detached with accutase and collected by centrifugation at 800 ×*g*. After washing with DPBS, cells were incubated with 2 μg/mL anti-mouse CD29-APC for 30 min at 4°C and analyzed with a S3e flow cytometer (Bio-Rad). For cancer stem cell detection, 4T1 cells were treated with AldeFluor (Stemcell Technology) following the manufacturer's protocol, and incubated with 4 μg/mL IgG 3A2 then 5 μg/mL anti-human IgG-PE (Becton Dickinson Bioscience) in 50 mM Tris-HCl (pH 7.5, with 150 mM NaCl, 5 mM CaCl_2_, 400 μM ZnCl_2_) for 30 min at 4°C. Polyclonal human IgG (Sigma) was used as a negative control.

For tumor microenvironment analysis, 2 million tumor cells from IgG 3A2- or control IgG-treated FoxP3-GFP Balb/c mice were pre-blocked with 1 μg CD16/CD32 antibody (10 min at 4°C in the dark) and then labelled with 0.2 μg fluorophore-conjugated antibody (anti-CD4 (APC-Cy7) for CD4^+^ T cell, anti-CD8 (PercpCP-Cy5.5) for CD8^+^ T cell, IL2 (APC) and IFN-γ(PE) for activation status of CD4^+^ and CD8^+^ T cells, anti-F4/80 (APC-Cy7) for macrophages, anti-iNOS (PE) for M1 phenotype, anti-CD36 (APC), anti-CD206 (PE-Cy7), anti-CD45 (APC) for CD45^+^ T cells, anti-NK1.1 (PE) for NK T cells, anti-CD11b (APC-Cy7)/Ly6G (PE-Cy5)/CD62L (PE-Cy7) for neutrophils, anti-Gr1 (PercpCP-Cy5.5)/CD 11b (APC-Cy7) for myeloid-derived suppressor cells, and anti-CD4 plus anti-CD25 (PE-Cy7) together with transgenic GFP fluorescence indicating FoxP3 positivity for Tregs (all antibodies from eBiosciences) for 30 min at 4°C in the dark. Cells were then washed with 2 mL 2% BSA three times and resuspended in 200 μL 2% BSA for flow cytometric analysis (BD Canto II).

### Tumor stem cell analysis

Tumor stem cells were isolated by their ability to form spheres in serum free medium. Briefly, 6000 4T1 cells were cultured in ultralow attachment plates (Corning) in stem cell selection medium (DMEM/F12 with no serum, but 2% B27 and 20 ng/mL hEGF) [[41, 47]] with different concentrations of IgG 3A2 or control IgG for 7 days. The number of tumor spheres (with diameters greater than 50 μm) was counted. For the stem cell proliferation assay, tumor spheres without any treatments were produced as described above and then digested with accutase (5 min at 37°C) into a single cell suspension. Ten thousand stem cells were then grown in ultralow attachment plates with different concentrations of IgG 3A2 or control IgG for 5 days. The effect of IgG 3A2 or control IgG on the number of cancer stem cells was determined by hemocytometer. For epithelial to mesenchymal transition (EMT) marker or stemness examination, cancer stem cells were treated with 10 μg/mL IgG 3A2 or control IgG for 5 days. RNA extraction was performed using Trizol (Invitrogen) and converted to cDNA. Quantitative real time PCR (Biorad CFX system) of gene expression associated with stemness (*sox2*, *oct4*), an EMT signature (*E-cadherin, fibronectin, vimentin, twist-1, snail-2*), and *MMP14* was performed and results were normalized to *β-actin*. Primer sequences are shown in Supplementary Table 1.

### Statistics

For *in vivo* data, the Mann-Whitney test was used to compare the control IgG- and IgG 3A2-treated groups. For comparisons of more than two groups and data from *in vitro* studies, ANOVA with post-hoc tests (Turkey's HSD for equal sample size and Scheffe for unequal sample size) and Bonferroni correction were conducted. Two tailed analysis was used. All data were expressed as the mean ± SD and a p value <0.05 was considered significant.

## SUPPLEMENTARY MATERIALS FIGURES AND TABLES



## Abbreviations

MMPs: matrix metalloproteinases
mAbs: monoclonal antibodies
EMT: epithelial to mesenchymal transition
cd: catalytic domains
nTIMP-2: N-terminal domain of tissue inhibitor of metalloproteinase-2
PEI: polyethyleneimine
