# Supplementary material for: Use of a novel camelid-inspired human antibody demonstrates the importance of MMP-14 to cancer stem cell function in the metastatic process
Source: Oncotarget. 2018 Jun 29;9(50):29431–44. doi: 10.18632/oncotarget.25654 (PMC6047671; doi:10.18632/oncotarget.25654)
Supplement: Supplementary file 1 [file oncotarget-09-29431-s001.pdf]

Use of a novel camelid-inspired human antibody demonstrates the importance of MMP-14 to cancer stem cell function in the metastatic process

SUPPLEMENTARY MATERIALS

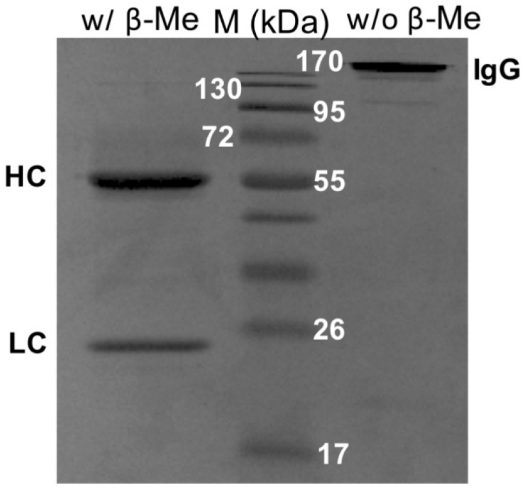

Supplementary Figure 1: IgG 3A2 expression and purification. IgG 3A2 was produced in 293F and purified with protein A. Purified IgG 3A2 IgG was analyzed w/ and w/o β-Me.

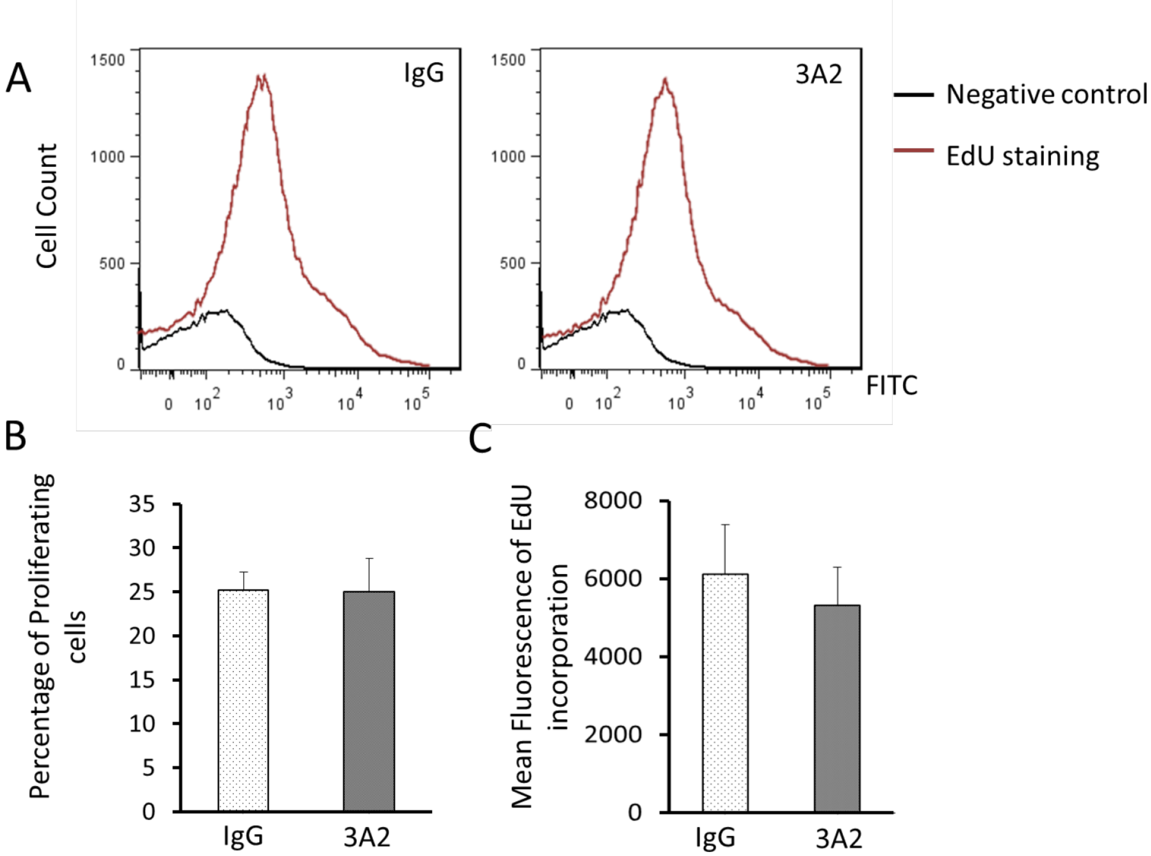

Supplementary Figure 2: IgG 3A2 treatment did not affect proliferation. The effect of IgG 3A2 on tumor cell proliferation was assessed by EdU incorporation **(A)**. Neither the percentage of EdU stained cells **(B)** nor the mean fluorescence intensity of EdU incorporation per cell **(C)** showed any difference between control IgG and IgG 3A2 treated group. (N=3) Data are presented as the mean ± S.D.

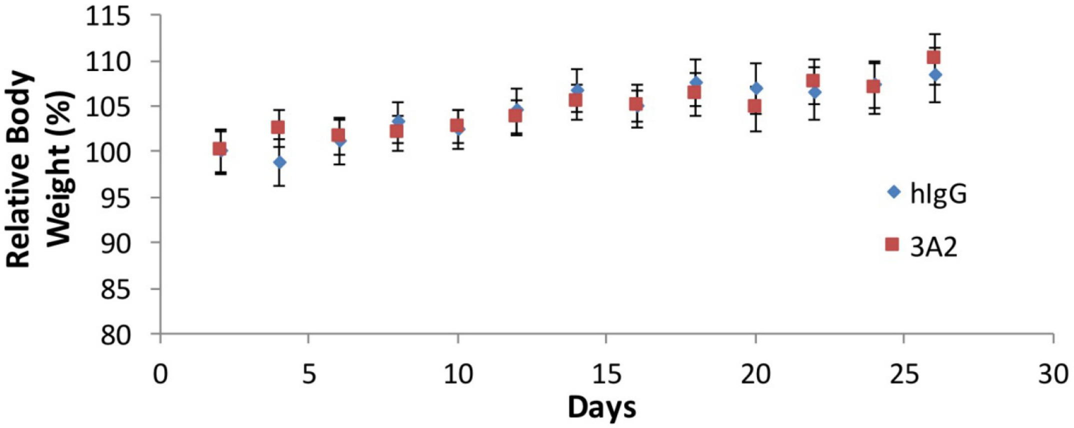

Supplementary Figure 3: No effect on body weight in mice receiving IgG 3A2 treatment. Animal body weight was assessed as one determinant of toxicity (n=11 in both control IgG and IgG 3A2 group). There was no observed effect on weight between control IgG and IgG 3A2 treatments. Data are presented as the mean ± S.D.

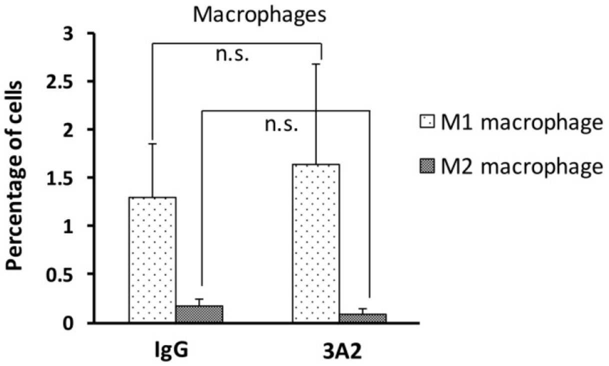

Supplementary Figure 4: A trend towards an increase in M1/M2 ratio by IgG 3A2 treatment. M1 (F4/80^{+}iNOS^{+}CD206^{-}CD36^{-}) and M2 (F4/80^{+}iNOS^{-}CD206^{+}CD36^{+}) macrophages in the primary tumors were examined. Variability does not allow a definitive statement to be made, but the trends toward an increase in M1 and decrease in M2 by IgG 3A2 (right) suggest the possibility. (N=3) Data are presented as the mean ± S.D.

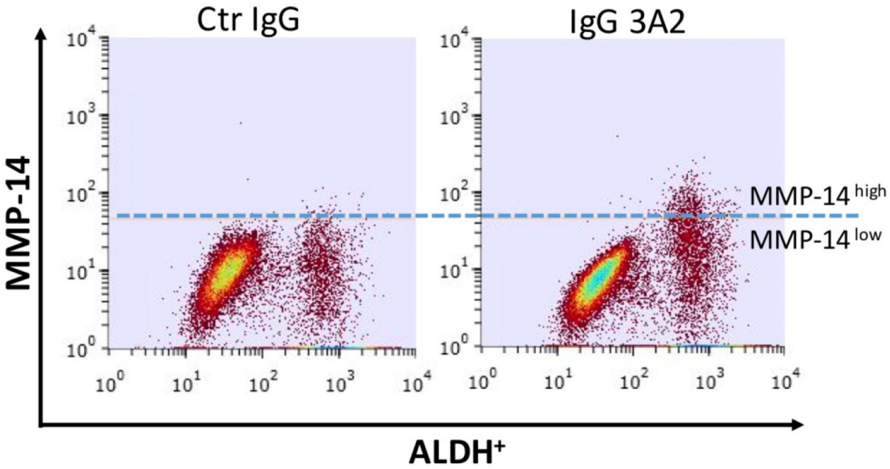

Supplementary Figure 5: MMP-14 expression levels on cell surface of bulk and stem cell populations. Polyclonal human IgG served as the negative control. Higher expression levels of MMP-14 were seen in cancer stem cells (ALDH^{+}).

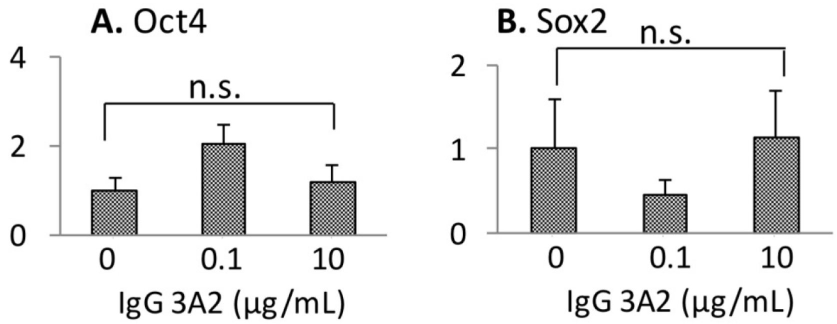

Supplementary Figure 6: Effect of IgG 3A2 treatment on the expression of 2 stemness markers in cancer stem cells. There was no significant change in *Oct4* **(A)** or *Sox2* **(B)** expression in 4T1 cancer stem cells between vehicle and 10 µg/mL IgG 3A2 treatment. Data are presented as the mean ± S.D.

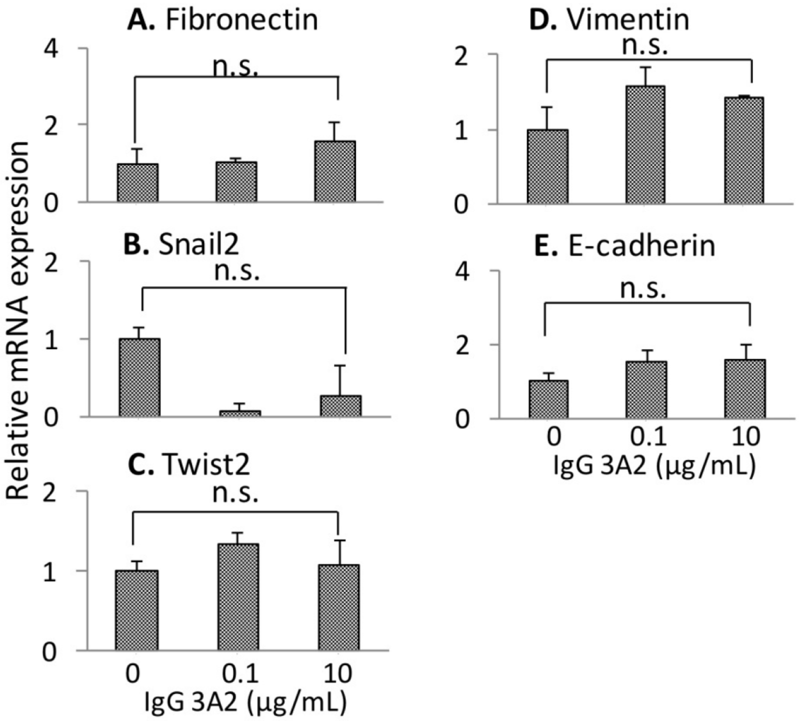

Supplementary Figure 7: Effect of IgG 3A2 treatment on the expression of epithelial to mesenchymal transition markers in cancer stem cells. There was no significant change in the expression of mesenchymal markers including *fibronectin* **(A)**, *snail2* **(B)**, *twist1* **(C)** and *vimentin* **(D)** in 4T1 cancer stem cells between vehicle and 10 μg/mL IgG 3A2 treatment, although there was a trend (not significant) towards a decrease in *snail2* with IgG 3A2. Also, no significant change was noted in the expression of epithelial marker, *E-cadherin* **(E)**, between vehicle and 10 μg/mL IgG 3A2 treatment. Data are presented as the mean ± S.D.

Supplementary Table 1: Primer list for qRT-PCR

Gene	primer sequence
sox2	F: TTAACGCAAAAACCGTGATG<br>R: GAAGCGCCTAACGTACCACT
oct4	F: TAGGTGAGCCGTCTTTCCAC<br>R: GCTTAGCCAGGTTCGAGGAT
E-cadherin	F: CGACCGGAAGTGACTCGAAA<br>R:TGTCCGCCAGCTTCTTGAAT
vimentin	F: ACTGCAGGAGCTGAATGACC<br>R: AAGGTCAAGACGTGCCAGAG
snail-2	F: CGCCTGGACCGTTATCCG<br>R: CTGCCGACGATGTCCATACA
twist-1	F: CCCACCCCACTTTTTGACGAAG<br>R: GCCAGTTTGATCCCAGCGTT
fibronectin	F: TGTGAGCCGGACAACTTCTG<br>R: CCTAGGTAGGTCCGTTCCCA
MMP-14	F: GAGTATGGGAGAGTGCCACG<br>R: GAACCATCGCTCCTTGAAGAC
actin	F: GACCCAGATCATGTTTGAGACC<br>R: AACGCAGCTCAGTAACAGTCC
